# Exploring the current engineering challenges of solid-state lithium-sulfur batteries with fundamental materials science: A review

**DOI:** 10.1557/s43581-026-00149-6

**Published:** 2026-02-12

**Authors:** Jun Yao Andrew Wong, James Moloney, Zhuangnan Li, Ziwei Jeffrey Yang, Yan Wang, Manish Chhowalla

**Affiliations:** 1 Department of Materials Science and Metallurgy, University of Cambridge, 27 Charles Babbage Road, Cambridge, CB3 0FS UK; 2 College of Chemical and Biological Engineering, Zhejiang University, 866 Yuhangtang Road, Hangzhou, 310058 China

**Keywords:** energy storage, ceramic, polymer, composite, ionic conductor

## Abstract

**Abstract:**

Lithium sulfur batteries offer high theoretical energy density and low material cost, but their practical use depends on electrolyte systems that satisfy several fundamental criteria. Solid state electrolytes provide a promising route by removing the liquid phase and improving safety. This review outlines the reaction pathways in lithium sulfur cells and the mechanisms of ion transport in solid electrolytes, followed by a generational comparison of major electrolyte classes, including polymers, oxides, sulfides, halides, garnets and composite systems. Four key criteria for solid state electrolyte use in lithium sulfur batteries are then examined: stability with electrodes, polysulfide behaviour, sulfur utilization and mechanical versatility. Existing strategies are evaluated in terms of how effectively they satisfy each requirement. Finally, two solid state cell designs that meet all identified criteria are proposed, together with quantitative considerations for electrolyte and cathode design. These insights provide a framework for guiding the development of practical solid state lithium sulfur batteries.

**Highlights:**

This work identifies the key requirements that solid electrolytes must meet to enable high-energy lithium–sulfur batteries. It also highlights two practical cell designs that satisfy these requirements and point toward viable solid-state lithium–sulfur technology.

**Discussions:**

1. The lack of quantitative data on polysulfide solubility in solid polymers remains a major barrier to rational design of solid-state lithium–sulfur batteries, raising the question of whether industry should invest heavily in this chemistry before these fundamentals are resolved.
2. Although solid electrolytes are widely promoted as a safer alternative to liquid systems, their real-world reliability against lithium dendrites remains unsettled, especially under manufacturing imperfections and high-power operating conditions.
3. The practical energy and sustainability benefits of solid-state lithium–sulfur batteries depend not only on materials breakthroughs but also on whether new manufacturing lines—potentially requiring ceramic processing and polymer casting hybrids—can be deployed at commercial scale without prohibitive cost or environmental burden.

**Graphical Abstract:**

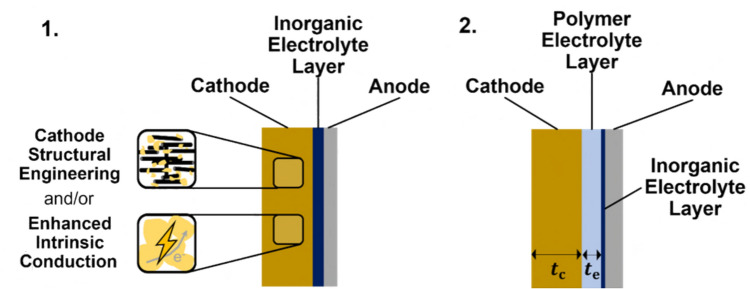

## Introduction

The high specific energy, long cycle life, and low self-discharge rates of lithium-ion batteries (LIBs) make them ideal for portable electronics and electric vehicles.^1–5^ However, for the large-scale electrification of transportation, batteries with higher energy densities are required.^6^ For instance, electric airplanes demand energy densities greater than 2000 Wh kg^−1^, which is approximately four times greater than the maximum reported theoretical energy density of state-of-the-art LIBs (340 Wh kg^−1^ – 580 Wh kg^−1^).^7,8^ Li–S batteries present a promising solution with a theoretical specific capacity of 1672 mAh g^−1^ and energy density of up to 2600 Wh kg^−1^.^9,10^ Additionally, Li–S batteries utilize earth-abundant and cost-effective materials with well-established supply chains, and reduce the reliance on particular critical minerals, such as cobalt and nickel, bringing economic and geopolitical advantages.^11^

One key issue with LIBs is the persistent risk of thermal runaway, which can lead to explosions and chemical fires.^12^ According to a report by Health & Safety International, the UK saw a 46% increase in LIB-related fires, from 630 in 2022 to 921 in 2023.^13^ Therefore, safer batteries that reduce the risk of chemical fires are essential.^14^ Replacing the flammable liquid electrolytes in traditional LIBs with solid, non-flammable electrolytes reduces this risk. Besides removing flammable components, solid electrolytes can act as physical barriers that block lithium dendrite penetration, which is a major cause of internal short circuits.^15,16^ Before short-circuiting occurs, dendrites concentrate the local electric field at their tips, accelerating ion transport, generating heat, and further promoting dendrite growth.^17,18^ This feedback loop leads to thermal runaway, posing substantial safety risks. By mitigating dendrite growth, solid electrolytes offer an important improvement in operational safety.

The focus of this review is the interplay of solid electrolytes with the Li–S battery chemistry. Currently, neither technology is widely used for commercial batteries, and many expect the successful fabrication of a solid-state Li–S battery system may solve key problems with each technology. Herein, the reaction mechanisms that occur within Li–S batteries are outlined and the ion transportation in solid electrolytes are described along with a brief overview of the advantages and disadvantages of different solid electrolyte types, and a comparison of the major generations of solid-state electrolytes (SSEs), ranging from early oxide and LISICON systems to modern LGPS and composite electrolytes, with liquid electrolytes included as a benchmark. After which, the criteria for solid state electrolyte utilization for Li–S batteries is discussed, focusing on stability with electrodes, lithium polysulfide shuttling, sulfur utilization, and mechanical versatility. Each of these are discussed in terms of the fundamental origins of the problem and solutions that are being explored to mitigate it. Finally, two battery structures are given which overcome all the presented challenges and metrics are provided to describe the required properties of these structures.

SSE research is a large and rapidly expanding field, with roughly 4700 publications in 2024, and only the most relevant discussions are included here.^19^ Zhao et al. provide a strong fundamental overview of solid electrolyte properties and behavior, while Boaretto et al. offer a comprehensive survey of promising materials and the practical considerations associated with commercializing solid-state batteries.^20,21^ Likewise, Li–S batteries, with approximately 2700 related publications in 2024, remain a highly active area of study.^19^ Much of the field is well summarized in the reviews by Zhao et al., Li et al., and Qian et al., which cover topics ranging from fundamental chemistry and materials science to the influence of operating conditions and long-term development strategies.^22–24^ For readers seeking a broader understanding of solid electrolytes and Li–S batteries outside of their intersection, these papers are recommended.

## The reaction mechanism of Li–S batteries

**Figure 1:**
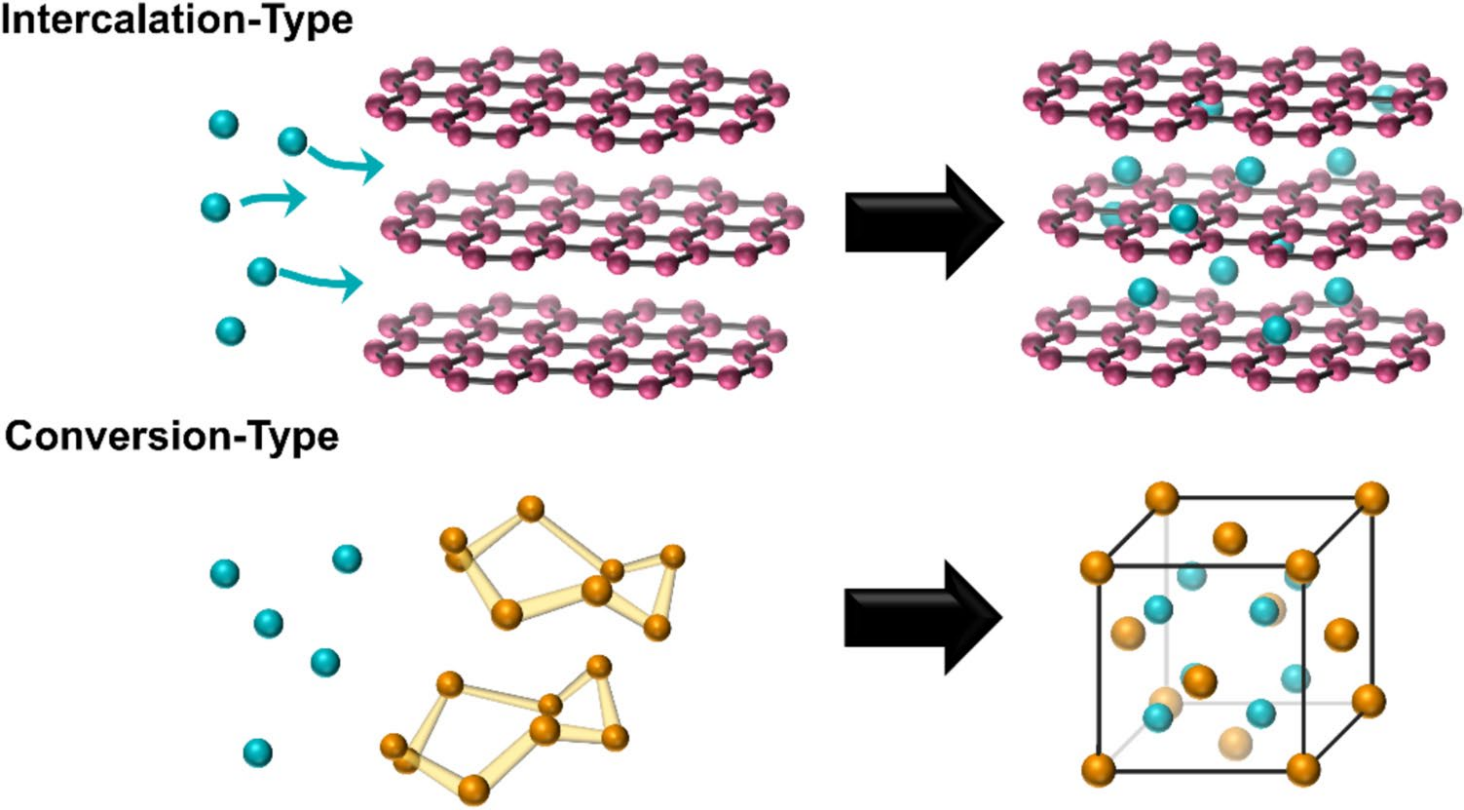
Comparison of Li ion storage in intercalation-type materials (e.g. intercalation of Li ions into graphite anode) wherein the structure is changed minimally and conversion-type materials (e.g. conversion of S_8_ cathode into lithium-sulfur compounds) wherein significant structural changes occur. (Pink spheres represent carbon atoms; blue spheres represent Li ions; gold spheres represent sulfur atoms.)

Li–S batteries store energy by utilizing a sulfur-based cathode that undergoes redox reactions that release or consume Li^+^ ions with accompanying phase transformations. This conversion-type mechanism is in contrast to the intercalation-type mechanism used by cathodes in traditional LIBs, wherein Li^+^ ions are accommodated into the electrode with minimal structural changes (Fig. 1). Commonly, cathode materials in LIBs are layered structures such as lithium cobalt oxide (LCO) and lithium nickel manganese cobalt oxide (NMC), although lithium iron phosphate (LFP) with a non-layered olivine structure is increasingly popular for its low cost. All these materials rely on intercalation which generally brings greater stability and longer cycle-life at the expense of energy density. A major challenge with Li–S batteries is improving the stability to be comparable to traditional LIBs.

In the charged state, the active material in the cathode of Li–S batteries is elemental sulfur. Upon discharge, it reacts with Li^+^ ions to form Li_2_S, however the conversion is highly complex and occurs via a series of intermediary reactions (see Eqs. 1–7). Since the overall reaction, wherein 2 mol of electrons are transferred per mole of S, usually occurs via a series of reactions, wherein a maximum of 1 mol of electrons is transferred per mole of S in a single reaction, the kinetic barrier for reaction is significantly reduced.^25,26^

Overall:

1 $$\mathrm{S}+2 {\mathrm{Li}}^{+}+2 {\mathrm{e}}^{-}\rightleftharpoons {\mathrm{Li}}_{2}\mathrm{S}$$

Intermediary reactions:

2 $$\mathrm{S}+\frac{1}{4}{\mathrm{Li}}^{+}+\frac{1}{4}{\mathrm{e}}^{-}\rightleftharpoons \frac{1}{8}{\mathrm{Li}}_{2}{\mathrm{S}}_{8}$$

3 $$\frac{1}{8}{\mathrm{Li}}_{2}{\mathrm{S}}_{8}+\frac{1}{12}{\mathrm{Li}}^{+}+\frac{1}{12}{\mathrm{e}}^{-}\rightleftharpoons \frac{1}{6}{\mathrm{Li}}_{2}{\mathrm{S}}_{6}$$

4 $$\frac{1}{6}{\mathrm{Li}}_{2}{\mathrm{S}}_{6}+\frac{1}{6}{\mathrm{Li}}^{+}+\frac{1}{6}{\mathrm{e}}^{-}\rightleftharpoons \frac{1}{4}{\mathrm{Li}}_{2}{\mathrm{S}}_{4}$$

5 $$\frac{1}{4}{\mathrm{Li}}_{2}{\mathrm{S}}_{4}+\frac{1}{6}{\mathrm{Li}}^{+}+\frac{1}{6}{\mathrm{e}}^{-}\rightleftharpoons \frac{1}{3}{\mathrm{Li}}_{2}{\mathrm{S}}_{3}$$

6 $$\frac{1}{3}{\mathrm{Li}}_{2}{\mathrm{S}}_{3}+\frac{1}{3}{\mathrm{Li}}^{+}+\frac{1}{3}{\mathrm{e}}^{-}\rightleftharpoons \frac{1}{2}{\mathrm{Li}}_{2}{\mathrm{S}}_{2}$$

7 $$\frac{1}{2}{\mathrm{Li}}_{2}{\mathrm{S}}_{2}+{\mathrm{Li}}^{+}+{\mathrm{e}}^{-}\rightleftharpoons {\mathrm{Li}}_{2}\mathrm{S}$$

**Figure 2:**
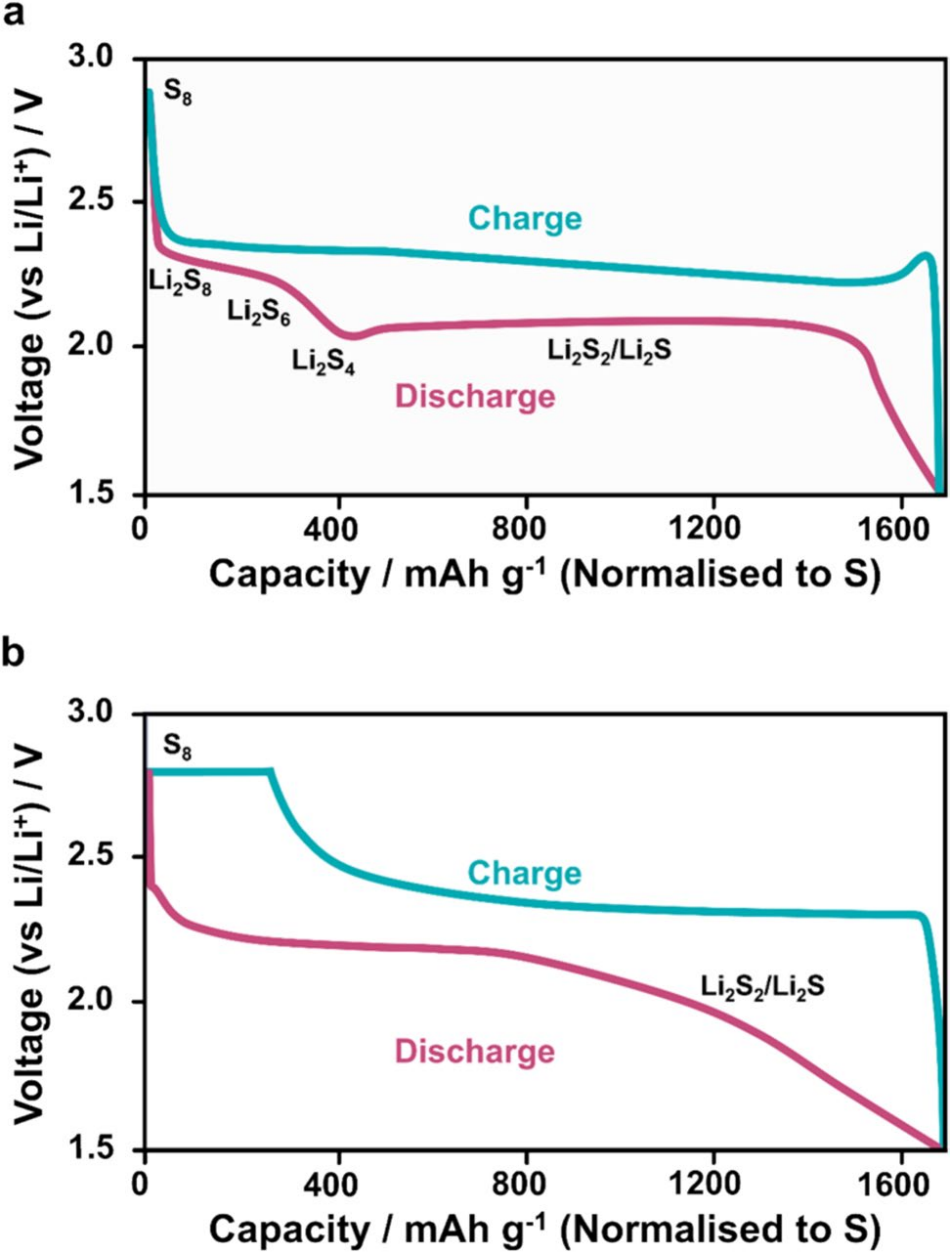
Typically observed voltage-capacity profiles for lithium-sulfur batteries with a liquid electrolyte (a) in which the intermediary lithium polysulfides can dissolve, and a solid electrolyte (b) with no or low solubility for lithium polysulfides. The 2.8 V plateau in (b) is due to constant current constant voltage charging. Adapted from Refs. 30, 31.

In a standard Li–S discharge profile with liquid electrolyte, as shown in Fig. 2a, the 2.3 V plateau corresponds to the conversion of S_8_ to Li_2_S_4_.^27^ The second voltage plateau at 2.1 V corresponds to the Li_2_S_4_ to Li_2_S conversion.^27^ The formation of Li_2_S_4_ from S_8_ requires the transfer of 0.5 mol of electrons per mole S, i.e. one-quarter the total capacity (418 mAh g^−1^_sulfur_)_._ The remaining reaction involves the formation of solid Li_2_S_2_ and Li_2_S. The initial nucleation of this solid phase is observed as a dip in the voltage profile upon discharging at the transition point between the end of the first step and the beginning of the second step (at approximately 418 mAh g^−1^_sulfur_ in Fig. 2a). In fact, the solubility of Li_2_S_4_ in the electrolyte is typically low and the observed voltage dip also likely arises from the nucleation of Li_2_S_4_ upon supersaturation of the electrolyte.^28,29^ On charging, the individual reactions are not distinguishable and so after overcoming the initial overpotential, the charge profile is generally an approximately linear slope.

With a solid electrolyte, the reaction mechanism varies depending on the electrolyte type and operating temperature. The lithium polysulfides generally have little or no solubility in the electrolyte and therefore the discharge profile does not appear as a two-step process, but rather as a single-step process with decreasing voltage. This arises when the sulfur-to-dilithium sulfide reaction occurs as a single, solid-to-solid reaction. As Li_2_S forms, it acts as an electronically and ionically insulating barrier that increases the internal resistance of the cell and reduces the measured voltage. If the length scale of the initial sulfur morphology is too large, the direct formation of Li_2_S will also restrict sulfur utilization and battery capacity because the internal resistance will increase dramatically prior to the completion of the reaction. This is a critical limitation of many solid-state Li–S batteries.

## Ion transportation in solid-state electrolytes

**Figure 3:**
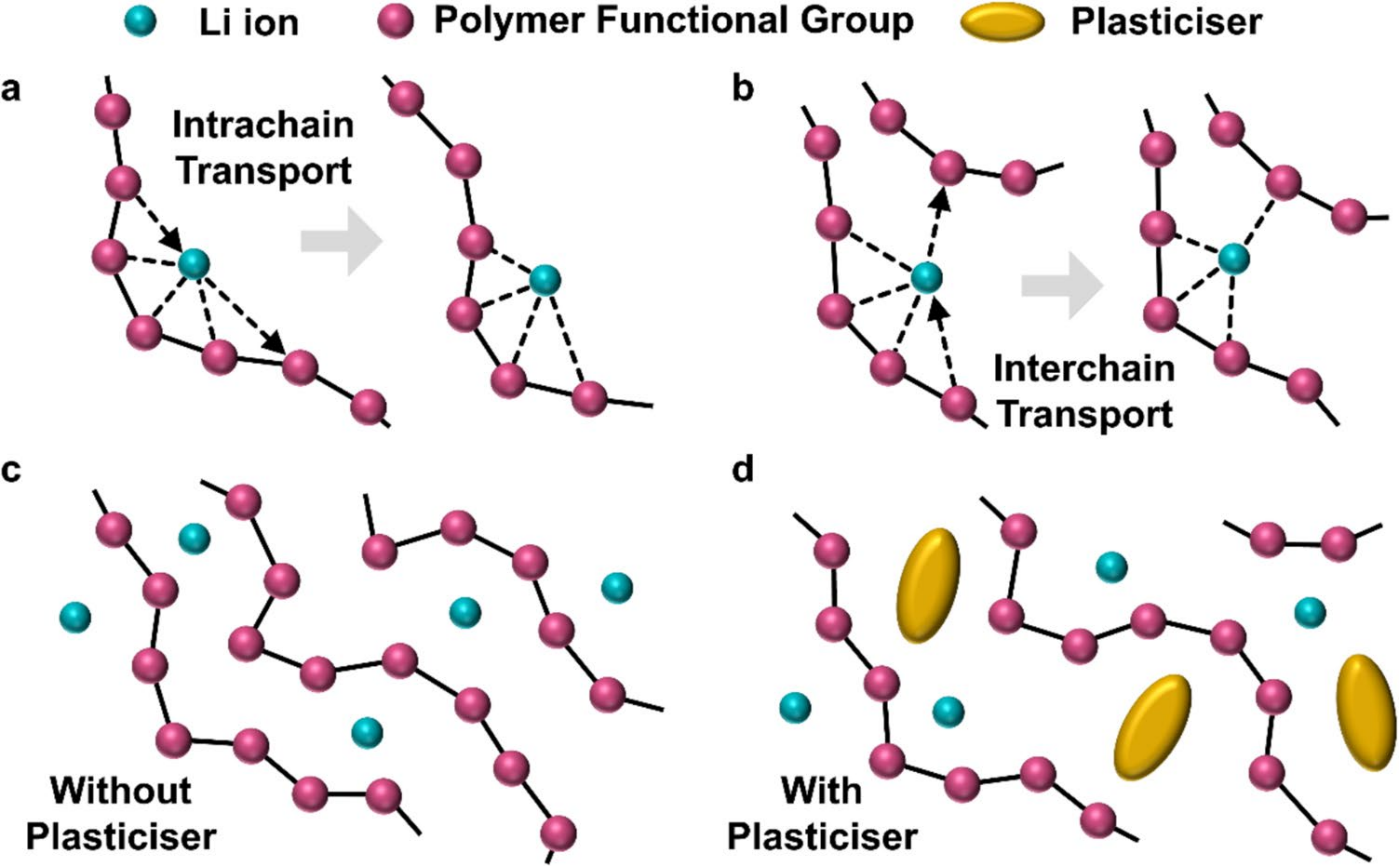
The successive breaking and forming of weak bonds between mobile ions and polar groups in polymers, accompanied with conformational changes in the polymer chains, allows for ion conduction in amorphous polymers. If the new bonds that form move the ion along the length of the polymer chain, it is described as intrachain transport (a), whereas if the ion moves between polymer chains it is described as interchain transport (b). In practice, both mechanisms will likely occur simultaneously and distinction between them is arbitrary. (c) shows a polymer without a plasticiser whereas (d) shows one with a plasticiser. The addition of the plasticiser typically increases the free volume around polymer chains, allowing greater space for conformational changes thereby decreasing the glass transition temperature and increasing ion conductivity.

In general, solid electrolytes are categorised as polymeric or inorganic.^32^ In polymeric solid electrolytes, ions are transported through amorphous regions, so efforts are often made to reduce the crystallinity of the polymer to increase ionic conductivity.^33^ There is weak electrostatic bonding between transporting ions (i.e. Li^+^ ions) and polar groups in the polymer chains. By breaking and reforming bonds with groups along the polymer chains or in different polymer chains, the ion is transported through the polymer. This is referred to as intrachain transport (see Fig. 3a) or interchain transport (see Fig. 3b), respectively.^34^ Typically, both mechanisms occur in an ion-conducting polymer, and any given ion is often bonded to multiple polymer chains at any given moment, experiencing both mechanisms simultaneously. Since these processes rely on conformational changes in the polymer, significant ionic transport only occurs at temperatures above the glass transition temperature of the polymer. Consequently, to achieve reasonable ionic conductivity at room temperature, the polymer electrolyte should have a low glass transition temperature. The most common choice of polymer is polyethylene oxide (PEO) with an intrinsic glass transition temperature of approximately -60 °C.^35,36^ The chosen polymer can be engineered in a multitude of ways to improve its properties.^37,38^

A Li salt must be dissolved in the polymer to enable Li^+^ ion conduction, and by choosing bulky salts the corresponding anion can act as a plasticiser, increasing the free volume around the polymer chains and thereby decreasing the glass transition temperature (see Fig. 3c & d).^39,40^ There is, however, a compromise to be made. If the glass transition temperature is lowered too much, the polymer may flow freely and no longer behave as a solid.^41,42^ The advantages of a low glass transition temperature and corresponding flexible polymer chains can be maintained whilst flow is prevented within meaningful timescales by introducing a small degree of cross-linking ^43^ or entanglement,^44^ or by hybridisation with insoluble, inorganic stabilizing materials.^45,46^ Research on solid polymer electrolytes has demonstrated ionic conductivities up to 0.114 mS cm^−1^ at 25 °C, lower than typical liquid electrolytes but sufficient for use in batteries.^47^ However, while the relative ease with which polymers can be processed industrially make them appealing for manufacturers,^48^ without the application of significant pressures in the order of 10^6^ – 10^7^ Pa during cycling,^49^ it remains unknown whether polymer electrolytes alone can prevent Li dendrite growth. The desire for an amorphous polymer with a high free volume fraction for good ionic conductivity often conflicts with the desire for a rigid polymer with high shear modulus for dendrite migration suppression. Again, cross-linking, chain entanglement or polymer-inorganic hybridisation may offer an answer but it is yet to be demonstrated.

**Figure 4:**
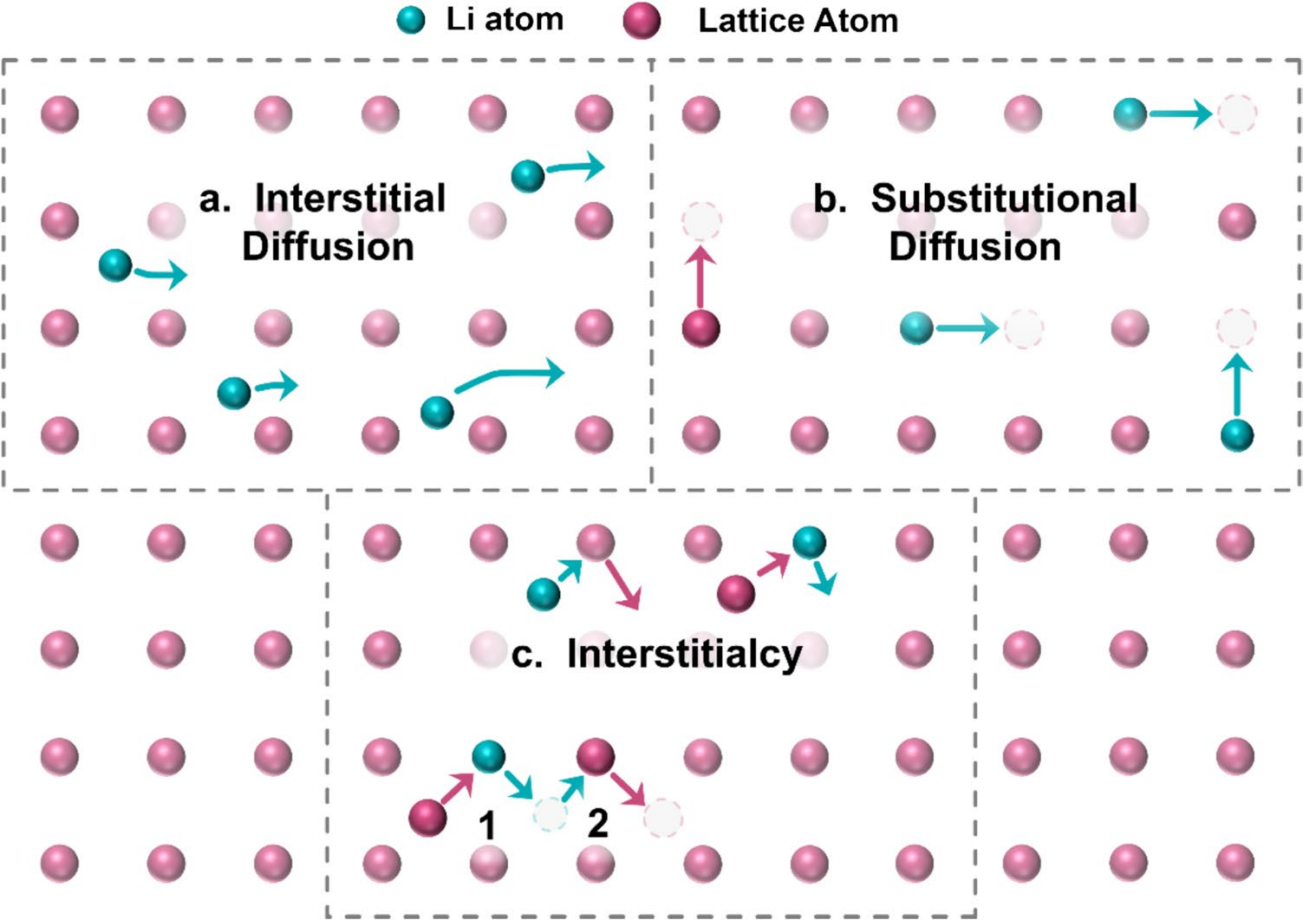
The most common ion conduction mechanisms in crystalline, inorganic electrolyte materials. In (a), interstitial diffusion, conducting ions are comparatively small enough to travel in the free space between atoms in the crystal lattice. In (b), substitutional diffusion, conducting ions occupy sites within the atomic lattice and vacancies in the lattice allow ions to hop between atomic sites. In (c), interstitialcy, an initial interstitial atom displaces a transporting ion from an atomic site in the lattice, which goes to displace another lattice atom in a different position. This results in a net movement of the conducting ion.

Ionic transport in inorganic electrolytes occurs via several different mechanisms depending on the atomic structure. In crystalline inorganic materials, ionic transport can occur via any/all of interstitial transport, substitutional transport, and/or interstitialcy (i.e. interstitial knock off). These mechanisms are shown in Fig. 4. Interstitial transport involves the movement of ions that are small enough to occupy and move throughout the spaces between atomic sites in the lattice. In substitutional transport, the transporting ions occupy atomic sites in the crystal and move via repeated ‘hopping’ to neighbouring vacant sites. In interstitialcy, both the conducting ion and lattice atoms can occupy interstitial sites and atomic sites in the lattice. As an interstitial atom/ion, each may knock the other out of an atomic site in the crystal. Upon repeated ‘knock off’ events, the conducting ion is transported through the crystal between atomic sites via movement as an interstitial ion.

Inorganic electrolytes may also be glassy (amorphous) or glass–ceramic in nature. In the amorphous regions of these materials, ionic transport occurs in a similar manner to that in polymers, wherein ions weakly bond to oppositely charged groups/ions in the amorphous matrix and successively break then reform these bonds.

While there are exceptions, each class of solid electrolyte brings differing advantages and disadvantages, which are summarised in Table 1 along with a brief elaboration. Comparing inorganic electrolyte materials to polymers, while inorganic electrolyte materials tend to have higher shear moduli enabling them to resist Li dendrite growth more effectively than polymers, the lower bulk moduli of polymers generally allow for lower interfacial impedance due to improved interfacial contact. In terms of stability, while some inorganic materials are more prone to oxidation, hydrolysis, and reaction with cathodes, polymers are generally more unstable with the Li anode.^34,50–52^

**Table 1:** Summary table of advantages and disadvantages for inorganic electrolyte materials and polymeric electrolyte materials.

Inorganic Electrolyte Materials	Advantages	High ionic conductivity (Sulfides and Halide-based SSEs can attain 10^–3^ to 10^–2^ S cm^−1^)	53, 54
		High transference numbers (Higher Li-ion selectivity vs. polymers)	55
		High Mechanical Strength (Resists Li dendrite growth)	56
		Large electrochemical stability window (Enabling the use in high voltage systems)	57
		Excellent thermal stability (Reduces flammability and risk of thermal runaway)	58
		Tunability of composition and structure (Conductivity, interfacial stability and processibility can be fine tuned)	59
	Disadvantages	Brittle (High speed of crack propagation in ceramics)	60
		High interfacial impedance (Due to poor solid–solid interfacial contact and lower ductility)	61
		High grain-boundary resistance (oxides)	62
		Sensitive to hydrolysis in air (sulfides and halides)	63, 64
		High cost (More expensive raw materials)	65
		Difficult processing (Harder to upscale production)	66
Polymeric Electrolyte Materials	Advantages	Excellent Interfacial Contact (Due to high malleability and ductility)	67
		Mechanical Processibility (Easy to shape and process)	66
		Light Weight (Reduces deadweight of electrolyte)	68, 69
		Safety (Reduced flammability versus liquid electrolytes)	70
		Tailorable Properties (Characteristics can be easily adjusted with additives and varying compositions)	71
	Disadvantages	Low room temperature ionic conductivity (Heavy reliance on segmental chain motion)	72
		Limited electrochemical stability window (Decomposes above 4 V)	73
		Poor mechanical strength (Susceptible to dendrite growth)	74
		Poor chemical lifetime (Prone to spontaneous degradation)	75
		Moisture sensitivity (Hydrophilic nature can lead to water entering the SSE)	76

Among the many performance metrics of SSEs, ionic conductivity is generally the most critical. Even if an SSE exhibits excellent thermal and chemical stability, low cost, and easy processability, it remains impractical for battery applications if its ionic conductivity is insufficient. Table 2 summarizes reported ionic conductivities for representative SSEs across different material generations.

**Table 2:** Compiled table various SSEs from different generations of SSEs and organic electrolytes, along with their respective references.

Name	Year	Ionic conductivity (S cm^−1^)	Testing temperature (°C)	Ref
*1st Generation Ceramic SSEs*				
0.5Li_3_PO_4_ – 0.5Li_4_SiO_4_	1977	1.6 × 10^–6^	25	77
Li_2_MCl_4_ (M = Mg, Mn, Fe, Cd)	1979	1 × 10^–2^–6 × 10^–3^	200	78
Li_4_GeO_4_ – Li_3_VO_4_	1980	1.59 × 10^–3^	25	79
Li – β – Alumina	1972	3 × 10^–3^	25	^80^
Li_3_N	1977	2–4 × 10^–4^	25	81
SiS – P_2_S_5_ – Li_2_S – LiI	1988	2.1 × 10^–3^	25	82
Li_3_N(H)	1983	6 × 10^–3^	25	81
β – Alumina (NaAl_11_O_17_)	1973	0.1–1.8 × 10^–1^	25	83
LISICON	1978	1 × 10^–6^	25	50
*Polymer SSEs*				
PEO-LiCF_3_SO_3_	1979	2.5 × 10^–5^	60	^84^
Li_3.3_PO_3.9_N_0.17_	1992	2.2 × 10^–6^	25	85
(PEO) complexed with LiClO_4_(TiO_2_)	2001	5.5 × 10^–5^	20	86
*2nd Generation Ceramic SSEs*				
0.03Li_3_PO_4_ – 0.59Li_2_S – 0.38SiS_2_	1992	6.9 × 10^–4^	25	87
Li_0.34_La_0.51_TiO_2.94_	1993	1 × 10^–3^	25	88
Li_7_La_3_Zr_2_O_12_	2007	1.87 × 10^–4^	25	89
Li_1+x_Al_x_Ti_2-x_(PO_4_)_3_	1997	1 × 10^–3^ to 1 × 10^–4^	25	90
Thio-LISICON (Li_4-x_Ge_1-x_P_x_S_4_)	2001	1 × 10^–2^ to 1 × 10^–3^	25	50
Li_7_P_3_S_11_	2007	1 × 10^–2^	25	91
[C_4_mpyr][NTf_2_]	2010	2.2 × 10^–3^	25	^92^
Li_6_PS_5_Cl	2008	1 × 10^–3^ to 1 × 10^–4^	25	93
*Organic liquid electrolytes*				
LiTFSI in EMI-TFSI	2004	2.7 × 10^–3^	25	94
PMMA-based polymer gel electrolytes containing NH_4_PF_6_	2006	1 × 10^–2^	25	95
*LGPS Generation*				
Li_9.54_Si_1.74_P_1.44_S_11.7_Cl_0.3_	2016	25 × 10^–3^	25	96
Li_10_GeP_2_S_12_	2010	10 × 10^–3^	25	97
Li_9.6_P_3_S_12_	2016	1 × 10^–3^	25	98
*Composite SSEs and 3rd Generation Ceramic SSEs*				
PEO + LiTFSI + wheat flour	2017	2.62 × 10^–5^	25	99
PMA + PEO	2019	2.05 × 10^−4^	65	100
PAN + PAN@LAGP + PEGDA	2019	3.7 × 10^−4^	25	101
PEO + SN + LiTFPFB + LITFSI	2019	0.5 × 10^−3^	30	102
P(PO/EM) + LITFPFB	2020	1.55 × 10^−4^	70	103
PS/PEG/PS	2019	1.1 × 10^−3^	70	104
Ca–CeO2/PEO	2020	1.3 × 10^−4^	60	105
LLZTO/PVDF	2021	2.73 × 10^−4^	25	106
PVEC – LITFSI – SiO_2_	2022	1.35 × 10^−3^	25	107
PVA – g – PCA – IL – HT	2022	4.78 × 10^−3^	60	108
PME–LiPVFM–LITFSI	2021	3.57 × 10^−4^	25	109
PPC – PCDF – LLZTO – LITFSI	2022	1.3 × 10^−4^	25	110
Li_3−x_Yb_1−x_Zr_x_Cl_6_	2021	1.1 × 10^−4^	25	111
PDOL + LiDFOB	2022	2.46 × 10^−4^	30	112
PVAC + TAGDA + AIBN	2022	1.02 × 10^−4^	25	113
PEO + LITFSI + LiI	2022	2.1 × 10^−4^	45	114
PVEC	2022	2.08 × 10^−3^	25	115
SiO_2_ – PVDF – HFP	2023	1.35 × 10^−3^	25	116
Li_6.55_Ge_0.05_La_3_Zr_1.75_Ta_0.25_O_12_	2023	6.61 × 10^−4^	25	117
Na_2.25_TaC_l4.75_O_1.25_	2024	2.5 × 10^−3^	25	118

In broad terms, and acknowledging notable exceptions, glassy and glass–ceramic inorganic electrolytes typically exhibit higher ionic conductivities than crystalline inorganic electrolytes, which in turn generally outperform polymer-based electrolytes. Within the inorganic class, sulfide and halide electrolytes currently demonstrate the highest conductivities, surpassing most oxide electrolytes, phosphate and nitride electrolytes, other emerging framework materials, and LISICON or NASICON systems.

## Solid-state electrolytes for Li–S batteries

When utilizing SSEs in Li–S batteries, SSE selection is not simple as there are significant considerations to be had.

## Stability with electrodes

One major challenge in Li–S systems is the decomposition of organic electrolytes on the lithium metal anode. Lithium’s high reactivity drives side reactions with organic electrolytes, leading to the formation of a solid-electrolyte interphase (SEI) layer.^119^ While the SEI can help suppress dendrite formation, these side reactions are typically uncontrolled and result in significant material loss. In some cases, they also pose safety risks, including internal gas evolution and overheating.^120^ For example, Camacho-Forero et al. conducted a density functional theory (DFT) analysis of DOL and DME decomposition and found that decomposition is enhanced at charged interfaces.^121^ Due to the presence of constant potentials and electric fields, activation barriers are lowered, making the reaction more thermodynamically favorable.

Therefore, SSE selection for Li–S systems needs to be carefully done to ensure that the SSE is relatively inert with lithium metal, to ensure the lack of uncontrollable side reactions. Composite SSEs have been shown to mitigate uncontrolled side reactions by being specifically engineered to form thin, stable SEI layers at the electrode interfaces, enhancing system stability. Zhao et al. demonstrated this using a composite SSE consisting of cross-linked poly(vinyl carbonate) on a Li_1.4_Al_0.2_Ti_1.8_Si_0.2_P_2.8_O_12_ (LATSP)-coated polypropylene separator.^122^ When paired with a LiFePO_4_/LiNi_0.8_Co_0.1_Mn_0.1_O_2_ cathode, the cell exhibited excellent cycling stability, retaining 81.56% of its capacity after 800 cycles at 2 C, decreasing from 141.11 mAh g^−1^ to 115.44 mAh g^−1^. Post-cycling analysis of a disassembled cell after 200 cycles revealed a completely smooth lithium anode with no dead lithium, indicating the formation of a stable SEI.

A key limitation of this study is its reliance on *in-situ* polymerization. Since no additional characterization, such as differential scanning calorimetry (DSC), was performed on the SSE, there is no direct physical evidence confirming complete polymerization within the cell. However, the authors demonstrated that the composite fully polymerizes outside the cell, which may provide sufficient support for this assumption. Another limitation is that Zhao et al. used a LIB cathode rather than a sulfur cathode. Despite this, the study validates that composite SSEs can be engineered to exhibit minimal reactivity with lithium, preventing uncontrolled side reactions. Extending this approach to sulfur cathodes remains a challenge. For instance, Chen et al. developed a composite SSE composed of PEO, LiTFSI, Li₆.₄Ga₀.₂La₃Zr₂O₁₂, and succinonitrile, which achieved a room-temperature ionic conductivity of 1.16 × 10⁻^4^ S cm^−1^.^123^ When combined with a sulfur-reduced graphene oxide cathode, the system demonstrated a specific capacity of 820 mAh g^−1^ after 100 cycles at 0.2 C. While this suggests short-term sulfur cathode stability, it does not establish long-term reliability. Additionally, while this study confirms compatibility with the sulfur cathode, it does not assess stability with the lithium anode. Further research is needed to develop SSEs that achieve sustained stability at both electrodes.

## Lithium polysulfide dissolution and shuttling

**Figure 5:**
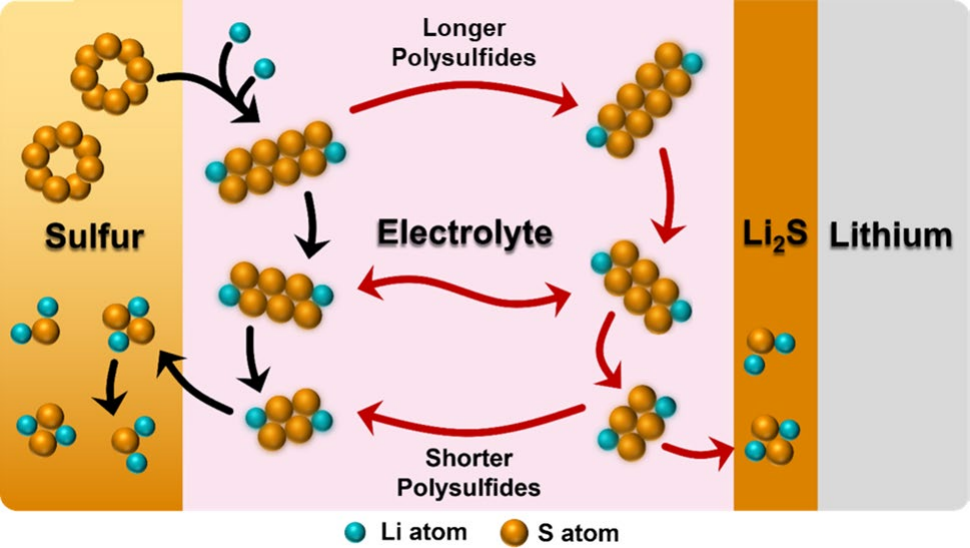
Schematic of the reactions and processes that are typically observed in Li–S batteries with liquid electrolytes during discharge. The desired reactions that contribute to cell capacity are indicated with black arrows whereas detrimental diffusion processes and unwanted reactions are indicated with red arrows. Diffusion of lithium polysulfide species, driven by concentration gradients, causes a loss of material from the cathode region and a resultant loss of capacity. Reduction reactions that occur at the anode, rather than the cathode, do not contribute to capacity. Note: although the lithium polysulfides are drawn as linear molecules, this is unlikely to be realistic, and their actual structure is an ongoing research matter.

During the discharge of lithium-sulfur (Li–S) batteries, elemental sulfur is reduced to dilithium sulfide (Li_2_S). Octoatomic α-sulfur, with a puckered ring molecular structure and orthorhombic crystal structure, is the most common form of elemental sulfur used for Li–S batteries due to its abundancy and thermodynamic stability. In cells with a liquid electrolyte, this initially reacts with Li^+^ ions to form Li_2_S_8_ and Li_2_S_6_. Since these are soluble in common liquid electrolytes, they dissolve, and a concentration gradient is generated across the electrolyte. Ideally, the lithium polysulfides would stay in the vicinity of the cathode and reduce fully to Li_2_S, however, the concentration gradient drives the diffusion of newly formed lithium polysulfides away from the cathode and towards the anode. These processes are schematically shown in Fig. 5. This concept is supported by the work of Coke et al., who used operando optical fluorescence microscopy to track lithium polysulfides in a liquid Li–S cell. During discharge, they observed increased fluorescence at the cathode as polysulfide concentration rose, followed by a gradual shift in brightness from the cathode toward the anode, indicating the migration of lithium polysulfides across the cell.^124^

At the anode, lithium polysulfides are reduced but this does not contribute to the capacity of the battery since the electrons that participate in these reactions have not passed through the external circuit. When larger lithium polysulfide molecules are reduced here, the formation of soluble, smaller lithium polysulfides may generate an opposing concentration gradient and counter-diffusion will occur to return active material to the cathode. When smaller lithium polysulfides are reduced at the anode, they form a layer of solid Li_2_S_2_ and Li_2_S on the anode. This represents a permanent loss of active material from the cathode and a corresponding loss of cell capacity. The ionically and electronically insulating nature of the layer also acts to increase internal resistance and therefore reduce cell voltage.

Cells that are experiencing lithium polysulfide shuttling will display a reduction in both cell capacity and cell voltage on every charge–discharge cycle and each contributes to a reduction in cell energy. For this reason, it is considered a critical issue in the Li–S battery field. Although less severe than in liquid Li–S cells, this remains a significant source of capacity loss in solid-state Li–S batteries. There have been many approaches investigated to prevent this and solid electrolytes offer a promising option. Inorganic solid electrolytes broadly have little or no solubility for lithium polysulfides. Moreover, the current understanding is that in these systems, direct solid–solid conversion between elemental sulfur and Li_2_S_2_/Li_2_S occurs with no intermediate formation of larger lithium polysulfides.^125^ This completely mitigates the problem of lithium polysulfide shuttling, but severely limits the achievable capacity, as discussed later in relation to sulfur utilization.

The direct conversion of S_8_ to Li_2_S was observed by Yang et al. using *in situ* TEM with only the native oxide of the Li anode as a solid electrolyte.^126^ Others have suggested that the same direct conversion occurs in all solid-state batteries wherein the electrolyte does not dissolve the lithium polysulfides, including crystalline inorganic solid electrolyte systems.^127,128^

Overwhelmingly in literature, solid polymer electrolytes are discussed similarly to solid inorganic electrolytes in the context of lithium polysulfide solubility, however there is an important distinction between them. Theoretically, solid polymer electrolytes may dissolve lithium polysulfides and therefore enable a different reaction mechanism more akin to that found in liquid electrolyte systems. In order to function, solid polymer electrolytes must already contain dissolved lithium salts, and since lithium polysulfides are also lithium salts, so as long as the electrolyte is not saturated, the dissolution of lithium polysulfides is possible. While the reaction is often described as a solid–liquid-solid conversion in liquid-electrolyte cells, the reaction with solid polymer electrolyte wherein significant lithium polysulfides can dissolve is more accurately described as a solid – solid-state solution – solid conversion. Solid-state solutions are a homogenous solid phase containing two or more components where components are dissolved or dispersed within the solid matrix, without forming separate phases. In general, solid-state solutions impose greater kinetic limitations on reactions than liquids but thermodynamically, this reaction would be similar to a solid–liquid-solid conversion and a two-step discharge plateau is expected. Indeed, at higher operational temperatures where kinetics are less limiting, two-step discharge is frequently observed with solid polymer electrolytes, unlike with solid inorganic electrolytes.^129–131^ However, to what extent polymer melting contributes to this effect is not fully explored in literature.

## Sulfur utilization

A key challenge in solid-state Li–S batteries is the reduced sulfur utilization compared to conventional Li–S batteries. With suppressed lithium polysulfide dissolution, Li_2_S readily forms at the sulfur/electrolyte interface early in the discharge process which creates an insulating layer and prevents further reaction.^126^ This direct solid–solid conversion of S_8_ to Li_2_S simultaneously requires 16 electrons and 16 Li^+^ ions per molecule of S_8_, resulting in a large kinetic barrier to reaction.^132^ The formation of electronically and ionically insulating Li_2_S during reaction only serves to further restrict the electrons and ions from reaching the sulfur. Without modifications, the size of sulfur structure is too large whilst the electronic conductivity of the sulfur and the ionic conductivity of the Li_2_S is too small. This leads to coring with remnant sulfur enclosed by Li_2_S_2_/Li_2_S and the prevention of full utilization of the sulfur.

Zhang et al. used nanoparticles of sulfur which reduces the effect of coring.^133^ They also used a mixture of carbon host materials with various dimensionalities (0D nanoparticles, 1D multiwalled nanotubes and vapour-grown fibers) to improve the conduction of electrons to the nano-sulfur particles. With this and a solid glass–ceramic electrolyte of 78Li_2_S-22P_2_S_5_, they achieved a specific capacity of 1141 mAh g^−1^ or ~ 70% sulfur utilization at approximately 0.1C rate, which approaches that achieved with liquid electrolytes.

Zhou et al. increased the intrinsic electrical conductivity of sulfur by 11 orders of magnitude to ~ 5.9 × 10^–7^ S cm^−1^ by doping the sulfur with iodine.^134,135^ They directly compared cells at various temperatures containing S_9.3_I to standard S-based cathodes. At 25 °C the standard cathode exhibited a capacity below 200 mAh g^−1^ (< 12% sulfur utilization) whereas the S_9.3_I cathode achieved a capacity of 812 mAh g^−1^ (49% sulfur utilization). Whilst this shows a dramatic improvement and demonstrates the principle that doping the sulfur can yield utilization increase, they found it was necessary to heat the cells to > 60 °C to achieve specific capacity values above 1000 mAh g^−1^. At these temperatures the cathode material would melt, according to their own differential scanning calorimetry data, and the discharge profiles display a two-step reaction, suggesting the reaction mechanism is no longer direct solid-to-solid. This is likely the cause of the high sulfur utilization at these temperatures.

As well as materials modifications, structural engineering can play a crucial role in increasing the sulfur utilization of solid-state Li–S batteries. Standard cathodes designed for liquid Li–S batteries are incompatible with solid-state systems. Cathodes designed for liquid-electrolyte systems assume the electrolyte can fill open porous structures in the cathode and therefore some cathode porosity aids in allowing Li^+^ ions to reach active material deeper in the cathode layer, thereby increasing active material utilization. In solid electrolyte systems, the electrolyte cannot usually fill pores within the cathode and so alternate methods to enable the Li^+^ ions to reach the active material must be explored.^136^ Ye et al. reviewed cathode film development for solid-state Li–S batteries and emphasized that enhancing ionic and electronic percolation is critical for achieving the theoretical capacity of sulfur.^137^ Reducing tortuosity by lowering cathode film porosity and minimizing binder content has been shown to be effective, as both factors create discontinuous percolation pathways that degrade performance. Alternatively, binders with improved ionic conductivities can be selected, thereby reducing the detrimental effect of the binder on the overall ionic conductivity of the cathode.^138^ For example, poly(3,4-ethylenedioxythiophene) polystyrene sulfonate (PEDOT:PSS) has an improved ionic conductivity in the range of 10^–3^ S/cm to 10^–4^ S/cm as compared to the more commonly used PVDF at 10^–7^ S/cm.^139–141^ In fact, polymer electrolyte materials are ideal for this if they have the additional property of being a good binder.

## Mechanical versatility

A key challenge for solid-state Li–S batteries is balancing mechanical toughness and ductility. The electrolyte must be sufficiently stiff to suppress dendrites, yet soft enough to maintain good interfacial contact and to accommodate for sulfur cathode expansion.

### Dendrite formation on the lithium metal anode

One major downside of liquid electrolyte Li–S batteries is safety, primarily due to the use of a pure lithium metal anode and flammable carbonate and ether electrolytes, which become explosive upon air exposure.^142^ The inherent surface roughness of lithium induces inhomogeneous current densities. In a COMSOL simulation by Vikalp et al., the local current density at the electrode edges was found to be twice as high as the overall cell current density (1 A cm^−2^ vs. 0.5 A cm^−2^).^143^ This inhomogeneity promotes dendrite formation and increases the risk of short circuits, leading to overheating, internal gas evolution, and mechanical failure.^17,18^

SSEs can mitigate dendrite formation by being specifically engineered for dendrite resistance.^15^ Zhang et al. demonstrated this using a layered composite solid electrolyte consisting of an I₂–PEO–lithium bis(trifluoromethanesulfonyl)imide (LiTFSI) layer and a Li₆.₄La₃Zr₂Al₀.₂O₁₂ (LLZAO)–PEO–LiTFSI layer.^16^ In the first layer, I₂ reacts with lithium to form LiI, which absorbs lithium dendrites and prevents them from piercing through the electrolyte. In a lithium symmetric cell, this electrolyte exhibited long-term stability over 2000 h at a current density of 0.2 mA cm^−2^, demonstrating effective dendrite suppression.

The main limitation of this study is that it was conducted in a lithium symmetric cell rather than a solid-state Li–S battery. However, since lithium dendrite formation is driven by anode interfacial processes, the concept is expected to be applicable in Li–S batteries as well. A broader challenge, however, is that while SSEs show promise under ideal lab conditions, they remain susceptible to dendrite growth in real-world applications due to inherent material imperfections, which can serve as nucleation sites for dendrites.^144^

### Interfacial contact

**Figure 6:**
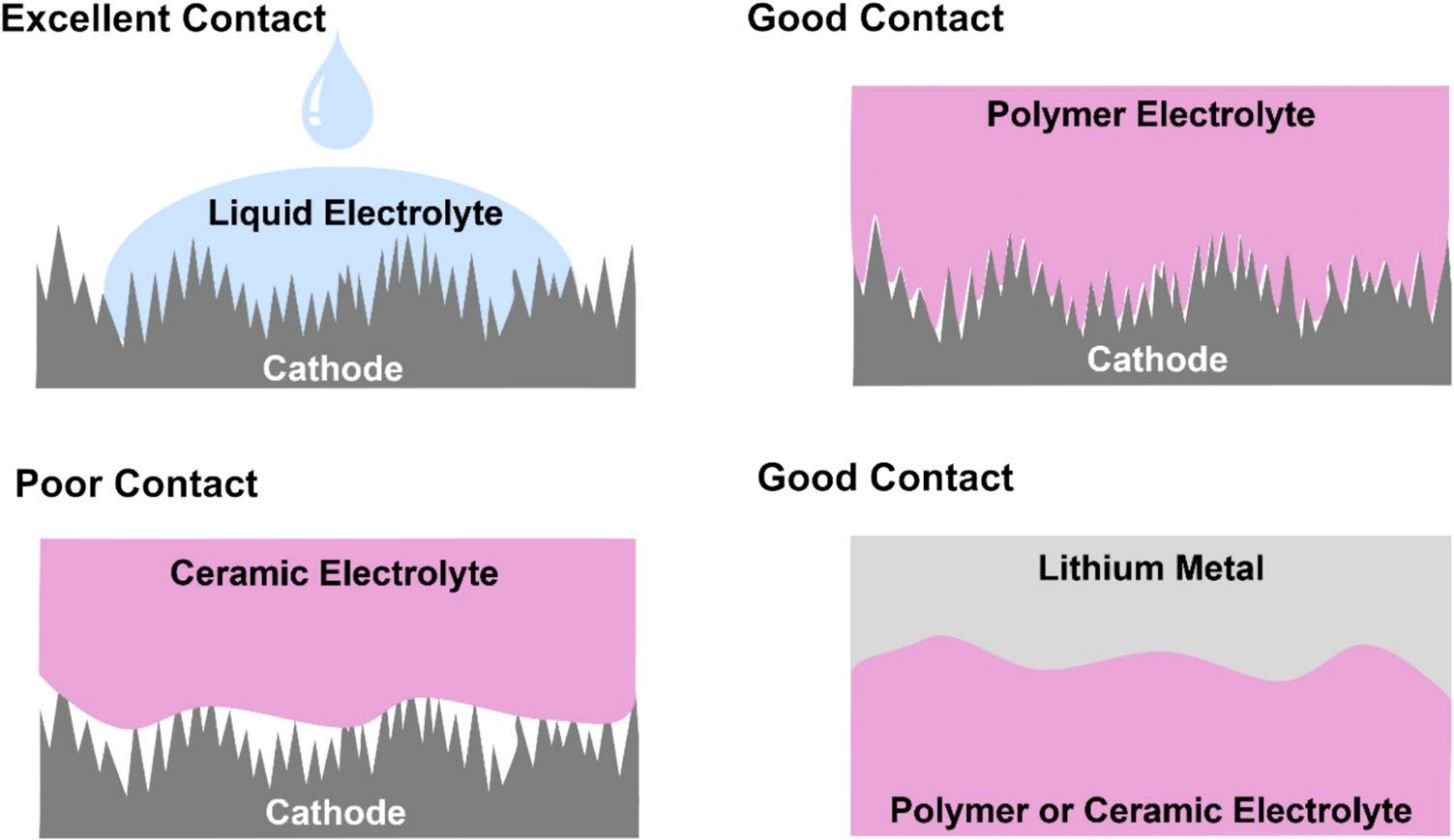
Comparison of the contact achieved between typical cathodes and various electrolytes, and also solid electrolytes with lithium metal.

In liquid electrolyte systems, the fluid nature of the electrolyte allows it to infiltrate porous electrodes and form molecular-scale contact at the interface, assuming the electrolyte is sufficiently wetting. This dramatically increases the effective contact area of the electrolyte–electrode interface thereby reducing the interfacial resistance.^145^ Many solid electrolytes, especially inorganic ceramic electrolytes, are rigid with bulk moduli up to 196 GPa.^146,147^ This not only prevents them from filling the pores of the electrodes but also limits their ability to conform to the surface of the electrodes (Fig. 6). Additionally, the roughness of the surfaces involved may cause the effective contact area to be far lower than the geometric contact area, increasing the interfacial resistance significantly.

Mechanically, polymeric solid electrolytes generally exhibit properties between liquid electrolytes and inorganic solid electrolytes. They have bulk moduli of the order of ~ 1 GPa,^148^ so they can deform at the interfaces and increase the effective contact area. By design, they do not flow readily but since they are usually operated above their glass transition temperature, limited movement at a molecular level is possible, which can enable further reduction in contact resistance via limited permeation of porosity.

There are several methods that can improve the interfacial contact in solid electrolyte systems. The discussion thus far, has assumed the components are assembled into a full cell in their dry form. If, however, one component is dried fully before the other is deposited as a slurry, better contact can be achieved because particles within the slurry are mobile and can therefore fill surface pores to varying degrees depending on surface roughness scale, particle size and particle mobility. In the specific case of casting a polymer electrolyte (or polymer-containing composite) electrolyte onto a dried electrode, either as a melt or as a solution, further enhancement can be achieved because the polymer molecules are free to make intimate, molecular-scale contact with electrode materials, unlike inorganic particles in electrolyte slurries which do not dissolve or melt. For example, Barcaro et al. demonstrated this in LIBs by tape casting a PEO based electrolyte directly onto a dried LiFePO_4_ (LFP) cathode, to produce an all-in-one cathode/electrolyte tape which possessed improved initial interfacial contact and cell performance.^149^ While the traditionally assembled LIB obtained an initial capacity of 12 mAh g^−1^, and only increasing to 66 mAh g^−1^ after 35 cycles, the cell assembled by tape casting obtained an initial capacity of 62 mAh g^−1^, and rose to 136 mAh g^−1^ within 35 cycles. This same principle can be applied to improving the interfacial contact between the SSE and the cathode. Even when polymer electrolyte membranes are assembled into a cell dry, sufficient heating of thermoplastic polymers can provide sufficient polymer mobility to enhance interfacial contact *in situ*. Heating steps prior to operation, often called ‘initialisation’, are frequently employed with solid polymer electrolytes for this reason.

Sharafi et al. applied the same principles to the anode-electrolyte interface where they studied the interfacial resistance in cells heated to 175 °C before cooling again.^150^ These cells used an LLZO solid electrolyte and lithium metal anode so at 175 °C, the lithium is soft enough to deform to the morphology of the LLZO and reduce interfacial resistance from 5822 Ω cm^2^ to a mere 2.7 Ω cm^2^ at 175 °C. Even after it was cooled back to room temperature, the interfacial resistance only increased back to 514 Ω cm^2^. This concept can be applied in polymer systems with low glass transition temperatures, as rubbery polymers form improved interfacial contact with the cathode over glassy polymers.

Another approach to improve interfacial contact is electrode modification. On the cathode side, solid polymer electrolytes can be incorporated directly into the electrode. Replacing conventional binders with ion-conducting polymers used in solid polymer electrolytes allows the same material to function in both the electrode and the bulk electrolyte.^151^ This creates an interconnected 3D network of ion- and electron-conducting pathways around the active material, similar to the pore-filling behavior of liquid electrolytes, and increases the effective contact area. On the anode side, coating lithium with a thin polymer layer can reduce interfacial impedance.^152^ Although this introduces an additional interface, the excellent conformality of polymers lowers the net interfacial resistance.^153^ Alloying the lithium surface can also improve wettability and surface smoothness.^154^ Alloy layers provide a smoother initial interface and promote more uniform lithium plating, which helps suppress dendrite formation.^155^ The drawback is that alloying reduces the specific capacity of the anode, since alloy layers can contain up to 60 wt percent of non-active metal.^156–158^

### Cathodic volume changes

**Figure 7:**
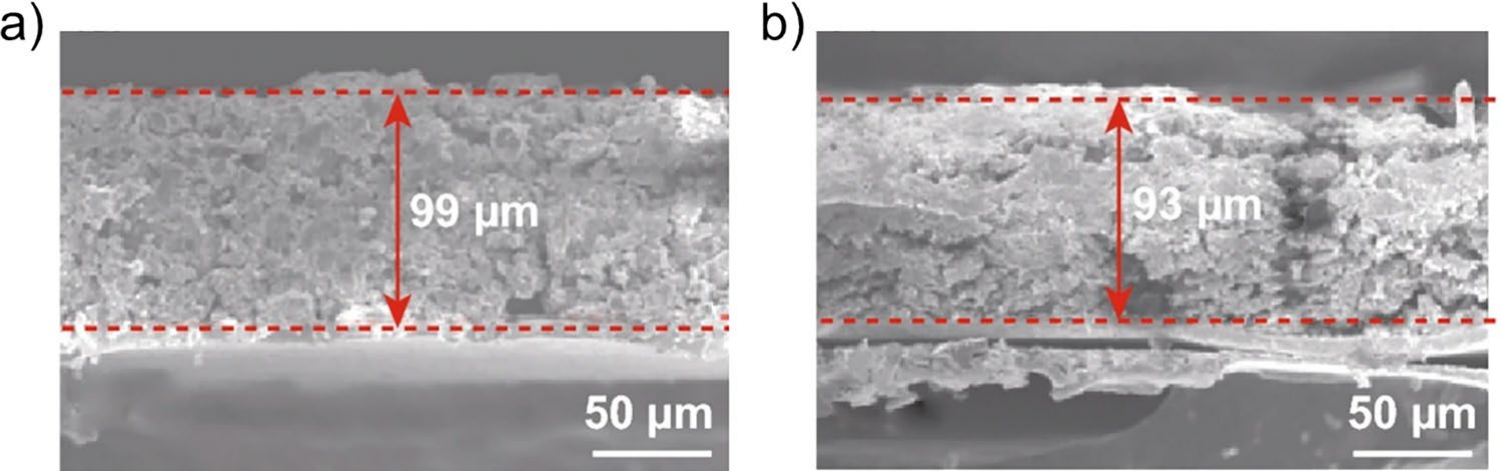
SEM micrographs of the cross section of a Ketjen black/sulfur cathode a pristine, b after 10 charge/discharge cycles between 1.7 V to 2.8 V at 2 C.^159^ Reproduced with permission.

The difference in density of elemental sulfur (2.07 g cm^−3^, 15.49 cm^3^ mol^−1^) and Li_2_S (1.66 g cm^−3^, 27.71 cm^3^ mol^−1^) causes a volume increase of up to 80% upon discharge. If this volume change cannot be accommodated, mechanical stresses are induced and cracking of the cathode material or delamination from the current collector may occur, especially on repeated cycling. This is shown in Fig. 7 and leads to loss of electrical contact in regions of the cathode and a resultant decrease in battery capacity on each cycle.

Generally, liquid electrolytes have low compressibility. For example, a common liquid electrolyte of lithium hexafluorophosphate in ethyl methyl carbonate and propylene carbonate has a bulk modulus of 1 – 3 GPa.^160^ In contrast, polymers such as variations of polyethyleneimine can be more compressible with a lower bulk modulus of 0.978 – 1.03 GPa.^148^ This enables pressure buffering by accommodating cathode volume changes. By absorbing the pressure increase from cathode expansion, solid polymer electrolytes reduce the rate of internal pressure buildup within the cell as compared to liquid electrolytes.^161^ This also allows solid polymer electrolytes to maintain consistent contact with the sulfur cathode throughout cycling, ensuring continuous electrical contact. Compared to liquid electrolytes, solid polymer electrolytes mitigate cathode cracking and enhance mechanical stability.

Luo et al. demonstrated this using a composite SSE composed of Li₆.₇₅La₃Zr₁.₇₅Ta₀.₂₅O₁₂ (LLZTO), polyimide film, and polyacrylonitrile.^162^ This composite SSE exhibited a high room-temperature ionic conductivity of 2.75 × 10⁻^4^ S/cm and a Li-ion transference number of 0.67. Paired with a sulfurized polyacrylonitrile cathode, the cell maintained 86.49% capacity retention from the 15th to the 1000th cycle at 1.0 C, achieving a specific capacity of 219.7 mAh/g with 98.9% coulombic efficiency. Post-cycling analysis revealed an indistinguishable interface between the electrolyte and cathode, demonstrating the mechanical stability of both the sulfur cathode and the composite SSE. The main limitation of this study is its low specific capacity, retaining only ~ 13% of the sulfur cathode’s theoretical 1672 mAh g^−1^, although it illustrates a good accommodation of cathode volume expansion and contraction over a large number of cycles.

**Figure 8:**
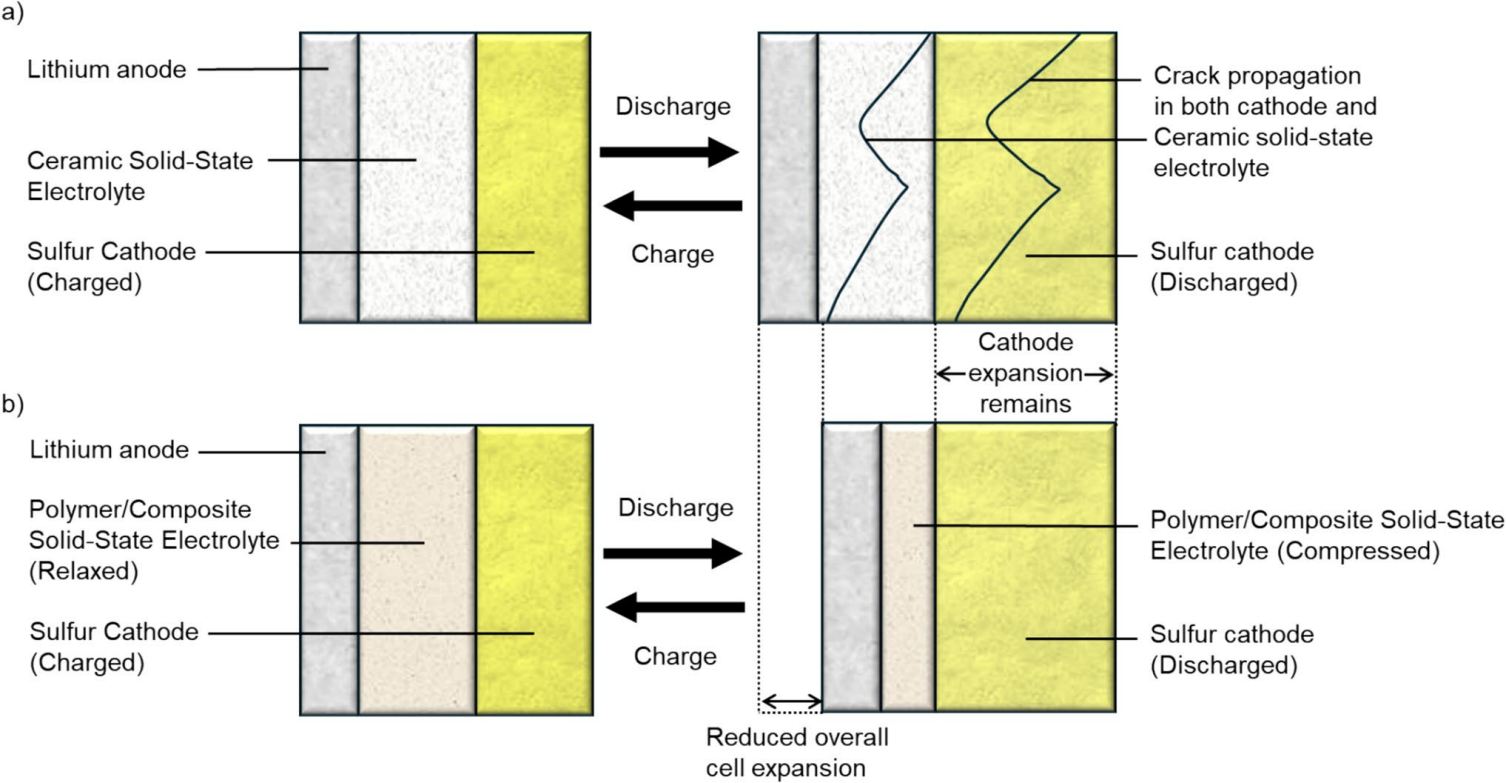
(a) Schematic of a solid-state Li–S cell employing an incompressible ceramic solid electrolyte. During discharge, sulfur cathode expansion induces repeated expansion–contraction cycles that can generate cracks in both the SSE and the cathode. (b) In a polymer/composite solid-state Li–S battery, the pressure-buffering capability of the SSE absorbs the volume change, reducing overall cell expansion and mitigating internal pressure buildup. The elastic nature of the polymer/composite SSE allows it to return to its original size during charging, maintaining continuous electrical contact with the cathode.

However, this applies only to polymer and composite SSEs. In contrast, inorganic SSEs with high bulk moduli cannot buffer pressure and may instead promote catastrophic failure (Fig. 8). Therefore, for systems where cathode volume change is significant, polymeric or composite SSEs offer better accommodation.

## Summary

Considering the challenges described previously, potential solutions to each are summarised below.

1. To prevent lithium polysulfide shuttling:
  Structural or chemical modifications to the sulfur host material to prevent lithium polysulfide movement by physical or electrostatic entrapment.

or

- Electron-insulating but ion-conducting layer between the electrodes which does not dissolve lithium polysulfides– i.e. an inorganic solid electrolyte layer or SEI. If this layer directly contacts the cathode, direct solid–solid reaction will occur (as typically observed with inorganic SSEs).

2. To enable full utilization of sulfur:
  Sulfur with morphology engineered to be small-scale such that direct solid–solid reaction goes to completion.

or

- Solid electrolyte layer at cathode that dissolves lithium polysulfides similarly to liquid electrolytes – i.e. solid polymer electrolyte.

3. To minimise interfacial resistance at the cathode:
  Use soft, ion-conducting material (i.e. a solid polymer electrolyte) at the cathode to deform to the morphology of the cathode

and/or

- Solid electrolyte layer at cathode that dissolves lithium polysulfides similarly to liquid electrolytes – i.e. solid polymer electrolyte.

4. To accommodate cathode volume changes:
  Integrate the electrolyte material into the cathode to increase contact area.

or

- High porosity cathode, but this reduces volumetric density, and increases ionic and electronic resistance unless structural engineering is used.

or

- Ion-conducting, low bulk modulus material at the sulfur cathode – i.e. a polymer electrolyte layer.

Lithium dendrite formation, though not specific to solid-state Li–S cells, remains a concern. Beyond alloyed lithium anodes, dendrites can be prevented by introducing a high–shear-modulus ion-conducting layer between the electrodes, which blocks dendrite growth without compromising the specific capacity of the anode.

**Figure 9:**
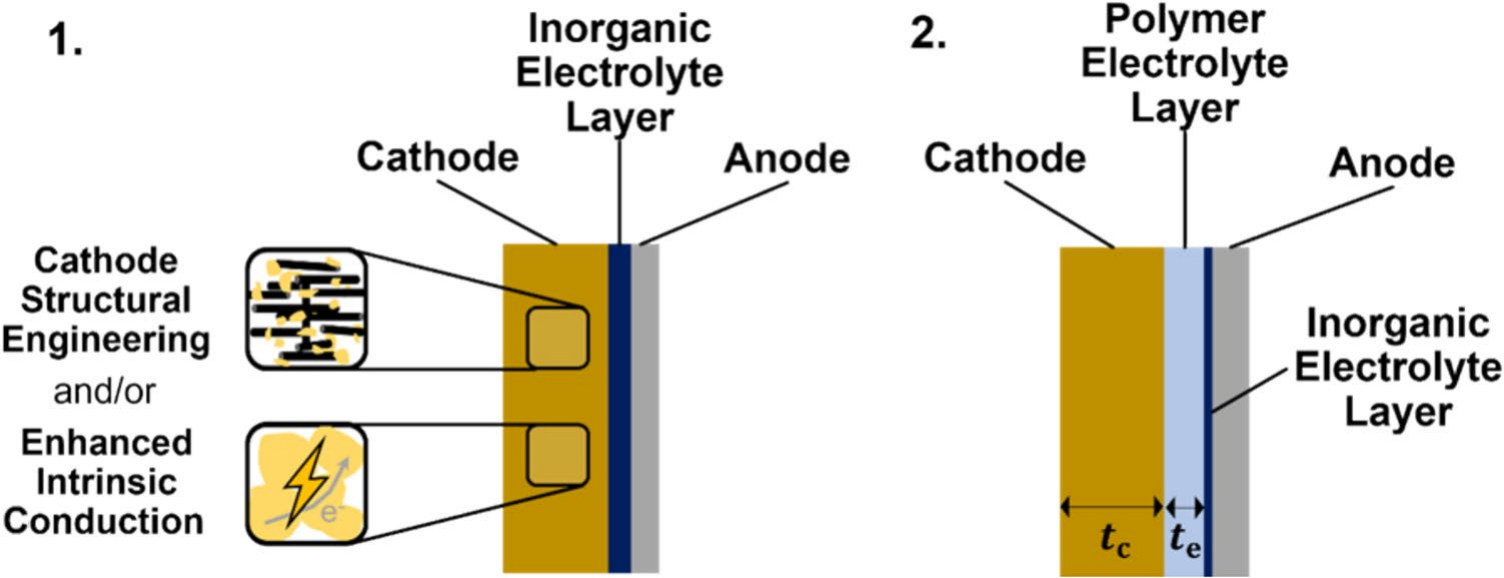
Two possible structures that satisfy the conditions set out to overcome the discussed challenges of solid-state Li–S batteries.

This review identifies two battery structures that currently show the most promise for solid-state Li–S systems. These designs, illustrated in Fig. 9, are discussed not as novel concepts but as promising directions with clear research gaps that still need to be resolved. The first uses a single inorganic solid electrolyte layer. This prevents lithium polysulfide shuttling but causes direct solid–solid reaction and may cause mechanical failure. To address this, the sulfur cathode would have to be engineered to either have sub-micron structure or enhanced electronic conductivity of sulfur and improved ionic conduction pathways to Li_2_S. Volume changes are accommodated by introduction of porosity or polymer electrolyte material into the cathode. Polymer electrolytes can also be used to improve the interfacial contact. Although some cathode engineering strategies have been reported, such as those by Cao et al. and Song et al., they focus mainly on catalytic enhancement and do not address the volumetric expansion of the sulfur cathode.^163,164^ This limitation is manageable at the lab scale, where cathodes are thin and expansion is small, but it will become a significant issue in pouch or cylindrical cells. Additional work in this area is therefore needed.

The second structure, which could be achieved with current technologies, and is mainly limited by the ionic conductivities of the electrolyte materials, uses a multilayer electrolyte. A solid inorganic electrolyte layer is required to prevent lithium polysulfide shuttling, but this should be as thin as possible, and may even be formed *in situ* as a thin inorganic solid electrolyte interface (SEI) on the anode. At the cathode, a solid polymer electrolyte is used to encourage two-step reaction and maximize sulfur utilization. This also ensures low interfacial resistance and some accommodation for volume changes. The thickness of this layer, ${t}_{\mathrm{e}}$, should be determined by the required volume to fully dissolve the lithium polysulfides during reaction.

If the solubility of Li_2_S_*n*_ in the polymer electrolyte is given by ${S}_{n}$, measured in moles per unit volume, a normalised solubility is defined:

$${C}_{n}=\frac{1}{n}{S}_{n}$$

For the lithium polysulfides that dissolve during cell cycling, (i.e. 4 < *n* < 8), the minimum normalised solubility is ${C}_{\mathrm{min}}$. Then for a cathode with thickness ${t}_{\mathrm{c}}$, areal sulfur mass loading of $\sigma$ and volume fraction $f$ which is polymer electrolyte,

$${C}_{\mathrm{min}}\left({t}_{\mathrm{e}}+f{t}_{\mathrm{c}}\right)=\frac{\sigma }{{A}_{\mathrm{S}}}$$

where ${A}_{\mathrm{S}}$ is the molar atomic mass of sulfur atoms, approximately 32.06 g mol^−1^.

Using approximate numbers of ${C}_{\mathrm{min}}=10 \text{mmol }{\mathrm{cm}}^{-3}$, $f=0.2$, $t_{c} = 100 \mu m$, $\sigma =5 \text{mg }{\mathrm{cm}}^{-2}$, a required polymer electrolyte thickness of $t_{e} \approx 140 \mu m$ is calculated. This is an order of magnitude thicker than the typical separator in liquid electrolyte cells which is the greatest drawback of this design. However, this approach also highlights a clear research gap, as quantitative studies on lithium polysulfide solubility in solid polymers are still lacking. Progress on this method will require new work in this area.

In parallel to the pathways for research discussed in this review, further work is needed to increase the ionic conductivity of solid electrolyte materials, and battery fabrication lines will need to change considerably for the adoption of SSEs to transpire. However, if a Li–S battery with an SSE of the correct structure and materials properties can be produced, it may enable the widespread adoption of both technologies. The use of solid electrolytes can make safer batteries and eliminate the issue of lithium polysulfide shuttling – a problem that has continually plagued the liquid-electrolyte Li–S battery community for over a decade in spite of extensive studies into methods to mitigate the problem. Conversely, the use of Li–S chemistry can provide the vast improvement to energy density with low cost, abundant raw materials that may make solid electrolyte batteries commercially viable.

## Data Availability

All data and material used in this review are appropriately referenced.
